# Supplementary material for: Curated and harmonised transcriptomics datasets of interstitial lung diseases
Source: Data Brief. 2025 Oct 14;63:112139. doi: 10.1016/j.dib.2025.112139 (PMC12581653; doi:10.1016/j.dib.2025.112139)
Supplement: Supplementary file 1 [file mmc1.zip › Supplementary_material/RNA-seq/fastQC reports/GSE184316/SRR15926795_fastqc.html]

SRR15926795.fastq.gz FastQC Report 

FastQC Report

Mon 5 Feb 2024  
SRR15926795.fastq.gz

## Summary

- Basic Statistics
- Per base sequence quality
- Per sequence quality scores
- Per base sequence content
- Per sequence GC content
- Per base N content
- Sequence Length Distribution
- Sequence Duplication Levels
- Overrepresented sequences
- Adapter Content

## Basic Statistics

| Measure | Value |
| --- | --- |
| Filename | SRR15926795.fastq.gz |
| File type | Conventional base calls |
| Encoding | Sanger / Illumina 1.9 |
| Total Sequences | 6958810 |
| Sequences flagged as poor quality | 0 |
| Sequence length | 25-381 |
| %GC | 51 |

## Per base sequence quality

## Per sequence quality scores

## Per base sequence content

## Per sequence GC content

## Per base N content

## Sequence Length Distribution

## Sequence Duplication Levels

## Overrepresented sequences

| Sequence | Count | Percentage | Possible Source |
| --- | --- | --- | --- |
| CAGTGGGGCCACGAGCTGAGTGCGTCCTGTCACTCCACTCCCATGTCCCT | 99648 | 1.4319689717063693 | No Hit |
| CACCTCCCCGAAGCTCAGGGAGAGCCCTGTTAGGGCCGCCTCTGGCCCTA | 93362 | 1.34163743513618 | No Hit |
| AGGCTACACACTGAGGACTCTGTTCCTCCCCTTTCCGCCTAGGGGAAAGT | 42142 | 0.6055920480656893 | No Hit |
| CAATCCACTTGATCCCAACTCATCTCTCATTTATTTCGGCTTCTTTTATT | 32863 | 0.47225028417215015 | No Hit |
| GGGAAGCGGGGAATGTCTACGTGCGTATGCACGTGGCACTCTCTGCCCGA | 32255 | 0.46351315814054417 | No Hit |
| CCCTGGCTGCTACAGGAGAATAGCAGACAGGTATAGTTAAGAAACAACTT | 32246 | 0.4633838256828395 | No Hit |
| AGCTGCTAAGTGCTGTGTTGTCGTTCCCCCTGCTTAAAATAAAGTTGTTT | 31019 | 0.4457515006157662 | No Hit |
| TTACAAAAGCAGAATAAAAGCGAAAAGAAATGAAAATGTTACACTACATT | 30922 | 0.4443575841271712 | No Hit |
| TTCAGCCCTGATCAAGACATGAGGGAGGCAGGGGCTCAGCTGAAGAAGCT | 30094 | 0.4324589980183393 | No Hit |
| TGATATGGTTCCTGGCAAGCCCATGTGTGTTGAGAGCTTCTCAGACTATC | 29106 | 0.4182611682169797 | No Hit |
| TGATGACACCCACCGCAACTGTCTGTCTCATATCACGAACAGCAAAGCGA | 26164 | 0.37598382482062304 | No Hit |
| AACAAGCACAAAGCCAAGAAAACCAACGATGCCAGATACTGAATTTCTAT | 21206 | 0.3047360108984151 | No Hit |
| CAGCAGTTTACACCTGCTAGGGTGTTCAAAGGTCAGTGCTATAGAAATTC | 21181 | 0.3043767540714576 | No Hit |
| GGCAATGAGCGGTTCCGCTGCCCTGAGGCACTCTTCCAGCCTTCCTTCCT | 19643 | 0.2822752740770333 | No Hit |
| GTCCACGTCACACTTCATGATGGAGTTGAAGGTAGTTTCGTGGATGCCAC | 17515 | 0.25169533296641233 | No Hit |
| TTTTCCATGAGCTTAATGATGCTTTCTCTGGGCTTTTGGGGGAGGGTGTC | 16800 | 0.24142058771542838 | No Hit |
| TCAGGAGACGCTCGTAGCCCTCGCGCTTCTCCTCGGCCAGTTCGCGGAAG | 14298 | 0.2054661644735235 | No Hit |
| AGACATCAAATCCTGCAGACAAGGGGAGCCCTCAGTCTGCAGGGCTCCAT | 14199 | 0.2040435074387719 | No Hit |
| TACCACTGCCACCTTCTCCATCGGCTCCACTGGCCTCGTGGTGTATGACT | 14073 | 0.20223285303090613 | No Hit |
| CTTCATGATGTAGCAGCAGGTGCCAGGGGCTGGCTTGTAGGCGATCAGCA | 12192 | 0.17520236937062514 | No Hit |
| CTCTCTTTCTGGCCTGGAGGCTATCCAGCGTACTCCAAAGATTCAGGTTT | 11257 | 0.1617661640424153 | No Hit |
| CAATGGGGTACTTCAGGGTCAGGATGCCACGCTTGCTCTGGGCCTCGTCG | 10757 | 0.15458102750326566 | No Hit |
| GAAACCCAGACACATAGCAATTCAGGAAATTTGACTTTCCATTCTCTGCT | 10685 | 0.1535463678416281 | No Hit |
| GGCATCAAAAGTGATGGACTGCCCATTGCTGGAGAAGACCTTCTCTCCTA | 10544 | 0.1515201593375879 | No Hit |
| TTTTTCCATGAGCTTAATGATGCTTTCTCTGGGCTTTTGGGGGAGGGTGT | 10250 | 0.14729529905256789 | No Hit |
| ACCTCTCTCTGGGCTTCTATTTCGACCGCGATGATGTGGCTCTGGAAGGC | 9870 | 0.14183459528281417 | No Hit |
| TCTTGGGTTATAATCATTGGCAATGTTGTAATCAACAACCCATGTTATTT | 9836 | 0.14134600599815197 | No Hit |
| GGAAAGGGGGCACTGACTGGTGATGCTGATATTGGAGTGCATCTGAGGCT | 9473 | 0.13612959687072934 | No Hit |
| GGAAGACAGTGTGGCGATTCCTCAGGGATCTAGAACTAGAAATACCATTG | 9461 | 0.13595715359378976 | No Hit |
| ACAAGAAGTACATCAAAGATTACATGAAATCAATCAAAGGGAAACTTGAA | 9039 | 0.12989289835474743 | No Hit |
| ATCAAAGAAGAACATGTGATCATCCAGGCCGAGTTCTATCTGAATCCTGA | 8766 | 0.12596981380437172 | No Hit |
| GGCAATGAGCGGTTCCGGTGTCCGGAGGCGCTGTTCCAGCCTTCCTTCCT | 8585 | 0.12336879437719955 | No Hit |
| GTCCACGTCACACTTCATGATGGAGTTGAAGGTGGTCTCGTGGATGCCGC | 8515 | 0.1223628752617186 | No Hit |
| GAAGGACCTCTTCAAGGAGAACTACAAACCACTGCTCAAGGAAATAAAAG | 8303 | 0.11931637736911915 | No Hit |
| CCAGTCTCGGCTCAGCCAAACACTGTCAGGGCCCCCAGCAGGGCCTTCAG | 7704 | 0.11070858379521786 | No Hit |
| CAATAAATAGGACACTTCTGCCAAAGGAACTCGTGCACAGCACCACAGAA | 7703 | 0.11069421352213955 | No Hit |
| TGTGGATGAATCTTGAACTGAGTTCCACTTGTAAACTTCTTGTTTCTTGT | 7658 | 0.11004755123361609 | No Hit |
| GACGAGTCAGAAAGGTCACGTCCTGCTCTTGGTCCTTGTCAGTGCCATGT | 7494 | 0.10769082644877499 | No Hit |
| ATTGATTCTTCCTACCCATGAGCATGGAATGTTCTTCCATTTGTTTGTAT | 7354 | 0.10567898821781309 | No Hit |
| CGTGAATACCAAGACCTGCTCAATGTTAAGATGGCCCTTGACATTGAGAT | 7302 | 0.10493173401774154 | No Hit |
| AGAGGGCTCCCAGAAGTGTGTGGCTGAGCTGGGTCCCCAGGCCGTGGGGG | 7210 | 0.10360966889453799 | No Hit |
| CGATCAGCACGGGCTCGTACTCTGCCACAAACTGATCACACTGCTTCTGG | 7176 | 0.10312107960987582 | No Hit |
| GGCAGACAAACCAGACATGGGGGAAATCGCCAGCTTCGATAAGGCCAAGC | 7136 | 0.10254626868674385 | No Hit |
| CAGTGGGCCACGAGCTGAGTGCGTCCTGTCACTCCACTCCCATGTCCCTT | 7094 | 0.10194271721745529 | No Hit |
| CAGCATGTTCCCTCTCCTCATGAGATTGGTGAAGAAAGTATTTGGCAAAG | 7040 | 0.10116672247122713 | No Hit |

## Adapter Content

Produced by FastQC (version 0.11.7)
